# The heterochronic LIN-14 protein is a BEN domain transcription factor

**DOI:** 10.1016/j.cub.2023.02.016

**Published:** 2023-03-27

**Authors:** Sharrell Greene, Ji Huang, Keith Hamilton, Liang Tong, Oliver Hobert, HaoSheng Sun

**Affiliations:** 1 Department of Cell, Developmental and Integrative Biology, University of Alabama at Birmingham, Birmingham, AL 35233, USA.; 2 Department of Biological Sciences, Columbia University, New York, NY 10027, USA.; 3 Howard Hughes Medical Institute.; 4 These authors contributed equally.

Heterochrony is a foundational concept in animal development and evolution, first introduced by Ernst Haeckel in 1875 and later popularized by Stephen J. Gould^1^. A molecular understanding of heterochrony was first established by genetic mutant analysis in the nematode *C. elegans,* revealing a genetic pathway that controls the proper timing of cellular patterning events executed during distinct postembryonic juvenile and adult stages^2^. This genetic pathway is composed of a complex temporal cascade of multiple regulatory factors, including the first-ever discovered miRNA, *lin-4,* and its target gene, *lin-14,* which encodes a nuclear, DNA-binding protein^2-4^. While all core members of the pathway have homologs based on primary sequences in other organisms, homologs for LIN-14 have never been identified by sequence homology. We report that the AlphaFold-predicted structure of the LIN-14 DNA binding domain is homologous to the BEN domain, found in a family of DNA binding proteins previously thought to have no nematode homologs^5^. We confirmed this prediction through targeted mutations of predicted DNA-contacting residues, which disrupt *in vitro* DNA binding and *in vivo* function. Our findings shed new light on potential mechanisms of LIN-14 function and suggest that BEN domain-containing proteins may have a conserved role in developmental timing.

To gain insights into the structural features of LIN-14, we first interrogated more than 100 recently sequenced nematode genomes for the presence of homologs of *C. elegans* LIN-14. BLAST sequence analysis readily recovered LIN-14 sequence homologs within the nematode phylum, but not outside. Sequence alignment of all 139 LIN-14 homologs defined a conserved 149 amino acid residues long region within a minimal LIN-14 protein fragment previously shown to bind DNA and to be sufficient to rescue *lin-14* mutant phenotypes (Figure 1A)^4,6^. Querying the AlphaFold structural database^7^, we found that this domain is predicted to be highly structured, primarily composed of several α helices (Figure 1B). Moreover, there are striking similarities with crystal structures of BEN domains from several different proteins, including *Drosophila insv* and BSG25A, and mammalian BEND family proteins (e.g., BEND3) (Figure 1B)^5,8^.

While BEN domains have not previously been described in *C. elegans,* the BEN domain is found in proteins across the animal kingdom, many of which are involved in transcriptional regulation and chromatin organization^5,8^. Since BEN domain proteins have been crystallized together with DNA target sequences^5^, we were able to predict that several basic, positively charged residues in the fifth (and last) α helix of the LIN-14 BEN domain contact DNA directly (Figure 1C). To experimentally confirm this prediction, we pursued both *in vitro* and *in vivo* approaches. In both types of analyses, we relied on our recent identification of genomic targets of LIN-14 protein, i.e., genes that were found to display *in vivo* binding to LIN-14, as assessed by ChIP-Seq analysis, and were transcriptionally dysregulated (i.e. derepressed) in *lin-14* mutant animals^9^. One such candidate is the neuropeptide-encoding gene *nlp-45.*

We expressed the predicted LIN-14 BEN domain in bacteria, both in its wild-type form and in mutated forms in which the four positively charged arginine residues in the fifth helix, predicted to be involved in DNA binding, are mutated to alanine residues. The arginine to alanine mutations did not result in protein destabilization as the mutant LIN-14 proteins were soluble and behaved similarly to the wild-type LIN-14 protein in gel filtration chromatography (Figure S1A,B in Supplemental information, published with this article online). Using gel shift assays, we found that the wild-type, but not the mutant LIN-14 proteins, bind to DNA sequences derived from the *nlp-45* locus (Figure 1D). The *in vitro* binding to the *nlp-45* promoter was sequence-selective, as shown in binding assays with single nucleotide mutations in the YGGAR motif (Figure S1C).

Next, we used CRISPR/Cas9 genome engineering to introduce the four arginine mutations into the endogenous *lin-14* locus. If these residues were indeed involved in DNA binding, we would expect such mutant animals to display the same defects as previously described in *lin-14* null mutant animals. We indeed found that *lin-14(syb5772)* Arg mutant animals are indistinguishable from *lin-14(ma135)* null mutant animals; they are sterile, have a dumpy appearance, a protruding vulva, display precocious alae, and show de-repression of *nlp-45* gene expression at the improper time, during the first larval stage (Figures 1E and S1D-F).

Structural homology searches using DALI against all predicted *C. elegans* protein structures from AlphaFold revealed several other *C. elegans* proteins with predicted similarities to the BEN domain (Figure S2). The only one previously characterized is SEL-7, a nuclear, DNA-binding protein with no previously known homolog that is, intriguingly, also involved in temporal patterning in *C. elegans*^10^. Another previously uncharacterized protein with two putative BEN domains, F12F6.1 (Figure S2), also shows temporally controlled expression during postembryonic development^9^.

The structural deorphanization of LIN-14 as a BEN domain-containing protein provides new vistas on both LIN-14 protein function as well as BEN domain proteins in general. Since many BEN domain proteins are involved in controlling chromatin architecture^5^, it is conceivable that, in addition to transcriptional regulation, LIN-14 may also play a role in chromatin organization. Since several non-nematode BEN domain-containing proteins have, like nematode LIN-14 and SEL-7, roles in temporal patterning^5,8^, such a function may have been the ancestral role of BEN domain proteins.

## Supplementary Material

MMC1

## Figures and Tables

**Figure 1.**
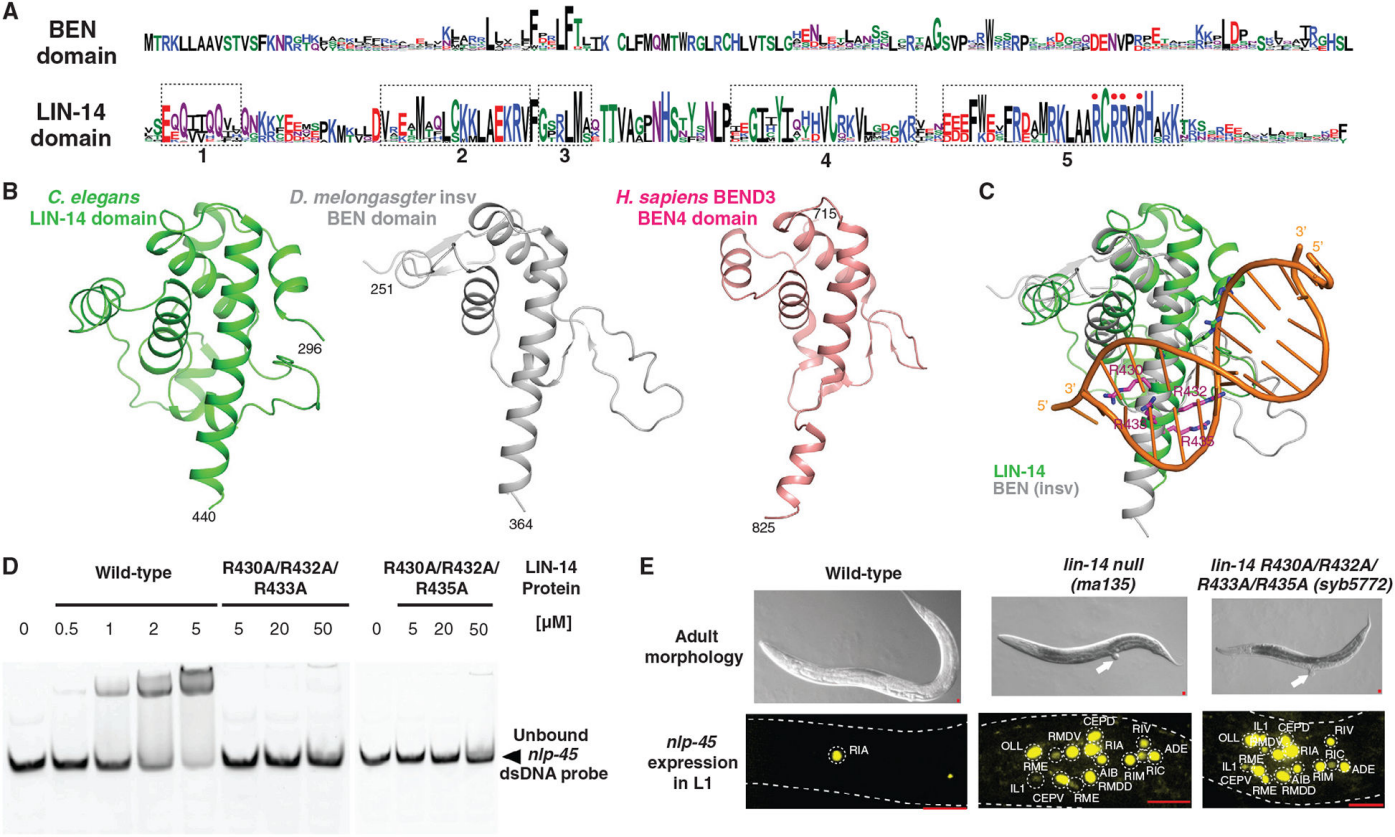
Structural homology of LIN-14 DNA-binding domain to BEN domain. (A) The motif logo of the LIN-14 DNA-binding domain is minimally similar to that of the BEN domain. The motif logos are generated using WebLogo 3. The alignment of 4,441 sequences from Pfam (PF10524) was used to generate the logo for BEN domains, while the alignment of 139 LIN-14 homologs across many nematode species (obtained from the WormBase ParaSite and aligned using ClustalOmega) was used to generate the logo for the LIN-14 domain. The dotted black boxes denote the five alpha-helices in the predicted LIN-14 structure (B,C), and the red dots denote the four arginine residues predicted to be important in DNA binding (C–E). (B) The predicted structure of the LIN-14 DNA-binding domain by AlphaFold (green, left, Uniprot Q21446) is homologous to the structures of BEN domains (*Drosophila melanogaster insv,* gray, middle, PDB 4IX7; *Homo sapiens* BEND3 BEN4, pink, right, PDB 7W27)^5,8^. See Supplemental Experimental Procedures in Supplemental information for how homologous structures were identified. (C) Predicted structure of the LIN-14 BEN domain (green) overlaid with the structure of *Drosophila insv* BEN domain (gray) bound to DNA (orange), showing the LIN-14 arginine residues (purple) that contact DNA that we mutate in panels D and E. (D) Wild-type LIN-14 proteins bind to the *nlp-45* promoter sequence while two separate arginine (R) to alanine (A) mutant LIN-14 proteins do not, as demonstrated in the electrophoretic mobility shift assay. The black arrow indicates unbound 6-FAM labeled *nlp-45* promoter sequence dsDNA probes. (E) Animals with mutation of arginine residues in LIN-14’s BEN domain, *lin-14(syb5772*), mimic *lin-14(ma135)* null mutant animals in adult morphology and the de-repression of *nlp-45* expression in first larval stage animals. Both *lin-14(ma135)* and *lin-14(syb5772)* adult animals are dumpy, egg-laying defective, sterile, and display a protruding vulva (indicated by white arrows, upper panel). The bottom panels show de-repressed *nlp-45* expression in the head of L1 *lin-14(ma135)* and *lin-14(syb5772)* animals compared to control animals. See Figure S1F for cell identification details. The red bars in the bottom right represent 10 μm.
