# Supplementary material for: The heterochronic LIN-14 protein is a BEN domain transcription factor
Source: Curr Biol. Author manuscript; Available in PMC 2023 Apr 7. (PMC10080584; doi:10.1016/j.cub.2023.02.016)
Supplement: MMC1 [file NIHMS1873816-supplement-MMC1.pdf]

## SUPPLEMENTAL INFORMATION

# The heterochronic LIN-14 protein is a BEN domain transcription factor

Sharrell Greene, Ji Huang, Keith Hamilton, Liang Tong, Oliver Hobert, HaoSheng Sun

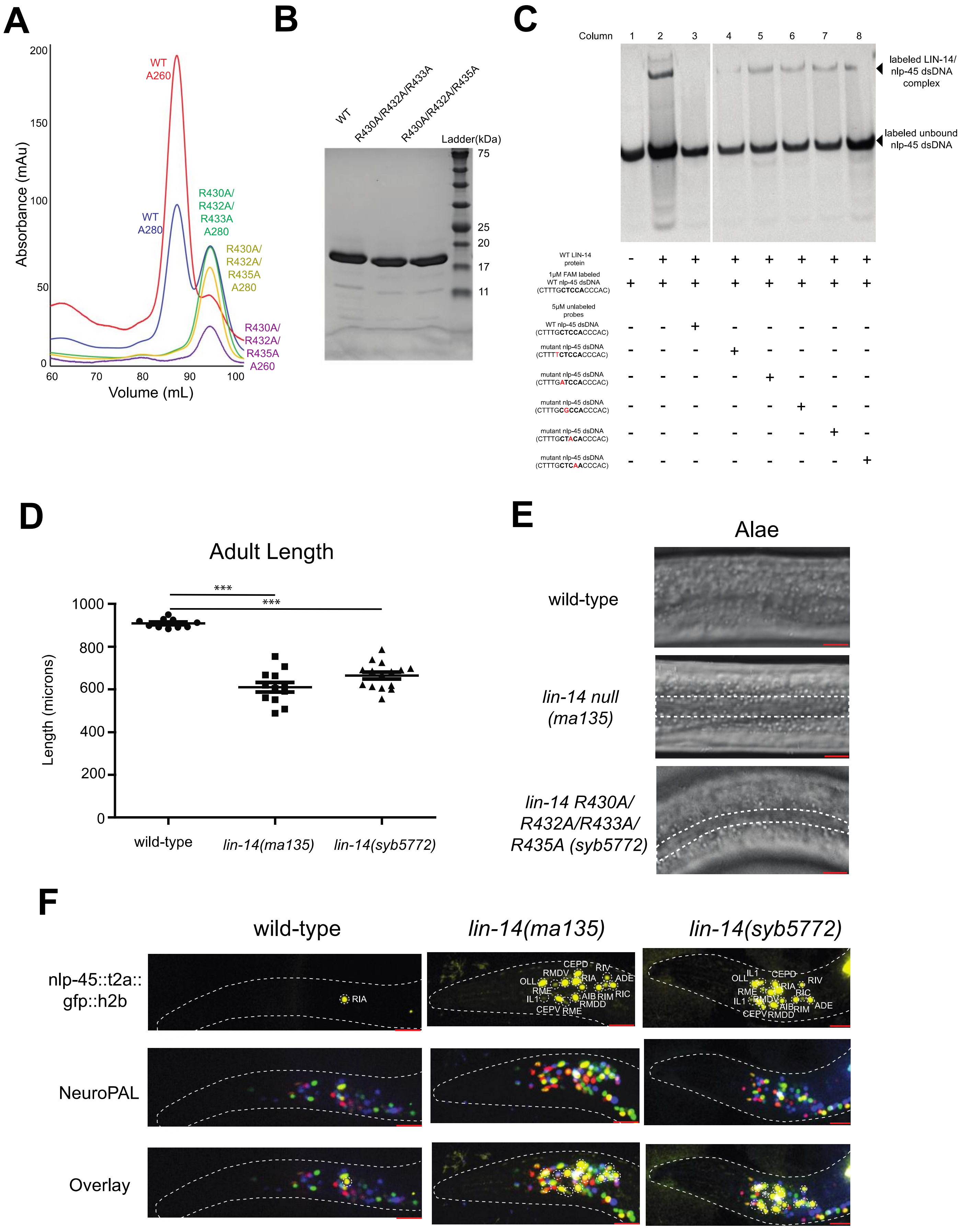

A
Absorbance (mAu)
Volume (mL)
WT A260
WT A280
R430A/ R432A/ R433A A280
R430A/ R432A/ R435A A280
R430A/ R432A/ R435A A260
B
WT
R430A/R432A/R433A
R430A/R432A/R435A
Ladder(kDa)
75
25
20
17
11
C
Column 1 2 3 4 5 6 7 8
labeled LIN-14/ nlp-45 dsDNA complex
labeled unbound nlp-45 dsDNA
WT LIN-14 protein
1μM FAM labeled WT nlp-45 dsDNA (CTTTGCTCCACCCAC)
5μM unlabeled probes
WT nlp-45 dsDNA (CTTTGCTCCACCCAC)
mutant nlp-45 dsDNA (CTTTTCTCCACCCAC)
mutant nlp-45 dsDNA (CTTTGATCCACCCAC)
mutant nlp-45 dsDNA (CTTTGCGCCACCCAC)
mutant nlp-45 dsDNA (CTTTGCTACACCCAC)
mutant nlp-45 dsDNA (CTTTGCTCAACCCAC)
D
Adult Length
Length (microns)
***
***
wild-type
lin-14(ma135)
lin-14(syb5772)
E
Alae
wild-type
lin-14 null (ma135)
lin-14 R430A/ R432A/R433A/ R435A (syb5772)
F
wild-type
lin-14(ma135)
lin-14(syb5772)
nlp-45::t2a:: gfp::h2b
NeuroPAL
Overlay
RIA

**Supplementary Figure S1: Characterization of LIN-14 *in vitro* and *in vivo.***

**(A, B)** Arginine to alanine mutations in the LIN-14 BEN domain disrupt nucleic acid binding but not overall protein stability.

**(A)** Gel filtration profiles for wild-type, and R430A/R432A/R433A and R430A/R432A/R435A mutants of LIN-14 BEN domain. Two peaks are observed for the wild-type LIN-14 BEN domain (A280 trace in blue). The first peak is contaminated by nucleic acids, as indicated by the A260 trace (red), while the second peak has no contamination, consistent with the BEN domain's DNA-binding property. The sample in the first peak was discarded. In comparison, the R430A/R432A/R435A mutant has only one peak (A280 in yellow, A260 in purple), at the same position and with the same profile as the second peak of wild-type BEN domain, with no nucleic acid contamination. The R430A/R432A/R433A mutant behaves the same as the R430A/R432A/R435A mutant (A280 in green, A260 trace not shown for clarity).

**(B)** SDS gel of purified samples of wild-type, and R430A/R432A/R433A and R430A/R432A/R435A mutants of LIN-14 BEN domain.

**(C)** Mutations of the YGGAR core motif in *nlp-45* dsDNA probe reduce LIN-14 binding affinity, as demonstrated in the competitive binding assay by EMSA. Incubation of wild-type (WT) LIN-14 (1µM) with FAM labeled WT *nlp-45* dsDNA probe (1µM) resulted in mobility shift ($2^{nd}$ column). The addition of 5µM unlabeled wild-type *nlp-45* dsDNA probes resulted in competition in LIN-14 binding and the lack of detected labeled LIN-14/*nlp-45* dsDNA complex (third column). Single nucleotide mutations of the YGGAR core motif as well as the adjacent G nucleotide resulted in reduced affinity to LIN-14, and reduced ability to outcompete LIN-14/labeled *nlp-45* dsDNA binding ($4^{th}$ to $8^{th}$ columns).

**(D-F)** *lin-14(syb5772)* mimics phenotypic effects of *lin-14(ma135)* null mutant.

**(D)** Length of wild-type (N2), *lin-14(ma135)* and *lin-14(syb5772)* young adult animals. Each animal is represented by a circle, rectangle or triangle. One-way ANOVA followed by pairwise t-test with Bonferroni correction: *** $P<0.001$.

**(E)** Ten out of ten *lin-14(ma135)* and ten out of ten *lin-14(syb5772)* L4 animals show precocious alae (dotted white box), whereas none of ten wild-type L4 animals have alae at this stage.

**(F)** *lin-14(ma135)* and *lin-14(syb5772)* animals show de-repressed *nlp-45* expression in L1 animals compared to wild-type animals. Representative microscope images of GFP expression (*nlp-45*), NeuroPAL for neuronal ID, and overlay are shown for control, *lin-*

*14(ma135)* and *lin-14(syb5772)* L1 larvae animals. Red bar in the bottom right represents 10 μm.

**A**

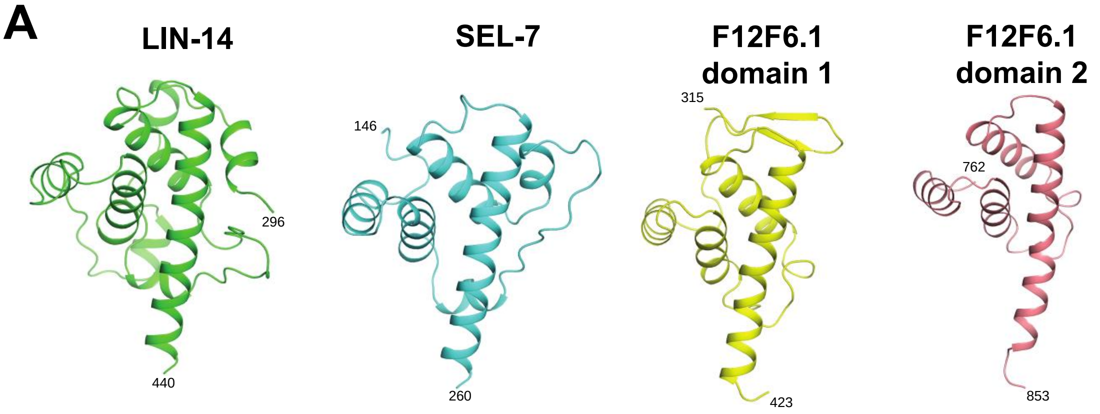


**B**

**Predicted 5th helix sequence**

| | |
|---|---|
| **LIN-14** | EEDFWDKFREAMRKLAA**R**C**RR**V**R**HA**KK** |
| **SEL-7** | TESQLSKAKLILSHLKNNA**K**V**R**NWTL**R** |
| **F12F6.1 domain 1** | KPTHVSKWKNCSFFIN**K**NM**R**EAVIAS |
| **F12F6.1 domain 2** | PLEGKQKWKNVTAAIN**R**NMTAAVVS**RR** |

**Supplementary Figure S2: Identification of additional BEN-domain containing proteins in *C. elegans*.**

**(A)** AlphaFold-predicted structure of BEN domains in LIN-14 (green, UniProt Q21446), SEL-7 (light blue, UniProt G5EGS7), and F12F6.1 (two separate domains, yellow and pink, UniProt G5EE62).

**(B)** Primary amino acid sequences of the predicted 5$^{th}$ helix of the BEN domains of LIN-14, SEL-7, a protein initially identified as a modifier of LIN-12/Notch signaling (Chen et al., 2004, *Genetics* 166, 151-160), and the uncharacterized F12F6.1 protein. As with LIN-14 (R residues functionally important in DNA binding and LIN-14 function bolded in red, other untested K residues bolded in black), positively-charged basic K/R residues are found in the carboxyl terminal of SEL-7 and F12F6.1 BEN domains (bolded in black), possibly important in binding to the negatively charged phosphate backbone of target DNA.

## SUPPLEMENTAL EXPERIMENTAL PROCEDURES

### *C. elegans* strains, handling, and construction

Worms were grown at 20 °C on nematode growth medium (NGM) plates seeded with *Escherichia coli* (OP50) bacteria as a food source. Worms were maintained according to the standard protocol. The wild-type strain used was the Bristol variety, strain N2. *lin-14(syb5772)* was generated using CRISPR-Cas9 gene editing by SunyBiotech. Since this strain is sterile, it was balanced with the same extrachromosomal array previously used for the *lin-14* null allele *ma135* (Ref 6) or the szT1 balancer. The strains used are as follows:

VT886: *lin-14(ma135); maEx167 [pVT333G (lin-14[1-3::4-13]::GFP); pVT301 (col-19::GFP)]*

PHX5773: *wt(+)/szT1[lon-2(e678); umnIs39] I;lin-14(syb5772)/szT1[umnIs40] X*

HUG60: *lin-14(syb5772); maEx167 [pVT333G (lin-14[1-3::4-13]::GFP); pVT301 (col-19::GFP)]*

OH16862: *nlp-45(ot1032[nlp-45::T2A::GFP::H2B]); otIs669; him-8(e1489)*

OH16845: *nlp-45(ot1032[nlp-45::T2A::GFP::H2B]); otIs669; lin-14(ma135); maEx167 [pVT333G (lin-14[1-3::4-13]::GFP); pVT301 (col-19::GFP)]*

HUG43: *nlp-45(ot1032[nlp-45::T2A::GFP::H2B]) X; otIs669; lin-14(syb5772); maEx167 [pVT333G (lin-14[1-3::4-13]::GFP); pVT301 (col-19::GFP)]; him-8(e1489)*

### Nematode lin-14 homology sequence alignment

*C. elegans* LIN-14 sequence was used as a bait to identify homologous proteins across all sequenced species in the Nematoda phylum, using the WormBase ParaSite BLAST function. After filtering out very short as well as duplicate sequences, all other sequences were aligned using ClustalOmega and visualized using Jalview2.

### Structural Prediction for LIN-14

The predicted LIN-14 structure was determined from AlphaFold (UniProt Q21446). The structural features examined here were taken from regions with confident or very high AlphaFold prediction scores and a high degree of conservation amongst nematode species. Proteins with similarity to the Alphafold-predicted LIN-14 structure were identified using DALI (Holm, L. DALI and the persistence of protein shape, 2020, Protein Sci 29, 128-140).

### Microscopy

Worms were anesthetized using 100 mM sodium azide and mounted on 5% agarose on glass slides. All images were acquired using a Nikon Ti2E inverted scope with a CREST v2 spinning disk unit and a Spectral3 light engine. Image reconstructions were performed using Nikon Elements software tools. Maximum intensity projections of representative images were shown. Colocalization with the NeuroPAL landmark strain (*otIs669*) was used for neuronal ID (Figure S4). Figures were prepared using Adobe Photoshop and Illustrator.

***Protein purification and expression***

*C. elegans LIN-14 (residues 292-441) and its mutants were over-expressed in E. coli and purified. A 6×His tag was fused to the N-terminal end of LIN-14. The proteins were over-expressed in E. coli* BL21 (DE3) Rosetta cells at 18 °C overnight. Cells were harvested the next day by centrifugation. The cell pellets were resuspended in lysis buffer containing 20 mM Tris (pH 8.0), 300 mM NaCl, 20 mM imidazole, 10 mM β-mercaptoethanol (βME) and 1× phenylmethylsulfonyl fluoride (PMSF). Two and a half cycles of sonication were performed to lyse the cell with each cycle consisting of 1 min processing time with 60% amplitude, 1 second on and 2 seconds off. The cell lysate was cleared by centrifugation at 12,000 rpm and 4 °C. Pre-equilibrated Ni-NTA beads (Qiagen) were incubated with the supernatant for 1 h at 4 °C, and the beads were washed thoroughly with lysis buffer before the target protein was eluted with 20 mM Tris (pH 8.0), 300 mM NaCl, 250 mM imidazole and 10 mM βME. Fractions containing LIN-14 were pooled and injected onto a pre-equilibrated gel filtration column (HiLoad 16/60 Superdex 200 prep grade, Cytiva) in the running buffer 20 mM Tris (pH 8.0), 250 mM NaCl, and 5 mM DTT. Purified LIN-14 and its mutants were concentrated and flash frozen in liquid nitrogen, and stored at –80°C.

**Site-directed mutagenesis**

The primers used to generate the mutants are as follows:

| | | |
|---|---|---|
| R430A/R432A/R433A | Forward primer | gcggcggcttgcgctgctgtgcgtcacgcgaag |
| | Reverse primer | cttcgcgtgacgcacagcagcgcaagccgccgc |
| R430A/R432A/R435A | Forward primer | cggcttgcgctcgtgtggctcacgcgaagaaaacc |
| | Reverse primer | ggttttcttcgcgtgagccacacgagcgcaagccg |

A modified QuikChange site-directed mutagenesis method was used. In the first stage, two reactions were performed separately for 11 cycles. One of the 25 μL reactions composed of 30 ng plasmid, 125 ng forward primer, 5 μL of 5× Q5 buffer (NEB), 0.66 U Q5 polymerase

(NEB) and 0.5 μL 10 mM dNTP, while the other had the same composition but contained the reverse primer. In the second stage, the two reactions were combined and another 0.66 U Q5 polymerase (NEB) was added. The elongation was performed for another 17 cycles. All mutated genes were sequenced.

### *Electromobility Shift Assays (EMSAs)*

Double-stranded DNA nlp-45FR were annealed by heating to 95 °C for 2 min, then cooled down on the laboratory bench. The forward strand nlp-45F (CTTTGCTCCACCCAC) with a 6-FAM label at the 5′ end was synthesized by Integrated DNA Technologies. The DNA at 1.0 μM concentration was mixed with various concentrations of LIN-14 wild-type and mutants in 20 mM Tris (pH 8.0), 250 mM NaCl, 1 mM $MgCl_2$ and 5 mM DTT in a total volume of 10 μL. The mixture was incubated for about 45 min at room temperature, and then 1 μL of 50% (v/v) glycerol was added and the samples were loaded onto a 15% (v/v) *polyacrylamide gel.* The electrophoresis was conducted at 170 V and 4 °C in 1x TBE (Tris, borate, EDTA) running buffer. The gel was visualized with a ChemiDoc (Bio-Rad) using Alexa Fluor 488 for FAM. For the competition assay, LIN-14 BEN domain (1 μM) and FAM-labeled wild-type nlp-45 dsDNA probe (1 μM) were mixed as described above. 5 μM of the unlabeled wild-type and mutant nlp-45 dsDNAs were incubated with the mixture before gel electrophoresis.

## AUTHOR CONTRIBUTIONS

S.G. and H.S. conducted the *C. elegans* experiments, J.H. and K.H. conducted the *in vitro* experiments, L.T. conducted the *in silico* structural analysis, L.T., O.H. and H.S. designed and supervised the project and co-wrote the manuscript. O.H. conceived this project.

## ACKNOWLEDGEMENTS

This work was funded the National Institutes of Health (R00HD098371 to H.S. and R35GM118093 to L.T.) and the Howard Hughes Medical Institute (O.H.).
